# Supplementary figures and images for: Developing the Breast Utility Instrument, a preference-based instrument to measure health-related quality of life in women with breast cancer: Confirmatory factor analysis of the EORTC QLQ-C30 and BR45 to establish dimensions
Source: PLoS One. 2022 Feb 4;17(2):e0262635. doi: 10.1371/journal.pone.0262635 (PMC8815914; doi:10.1371/journal.pone.0262635)

**S1 Fig:** Participant flow diagram

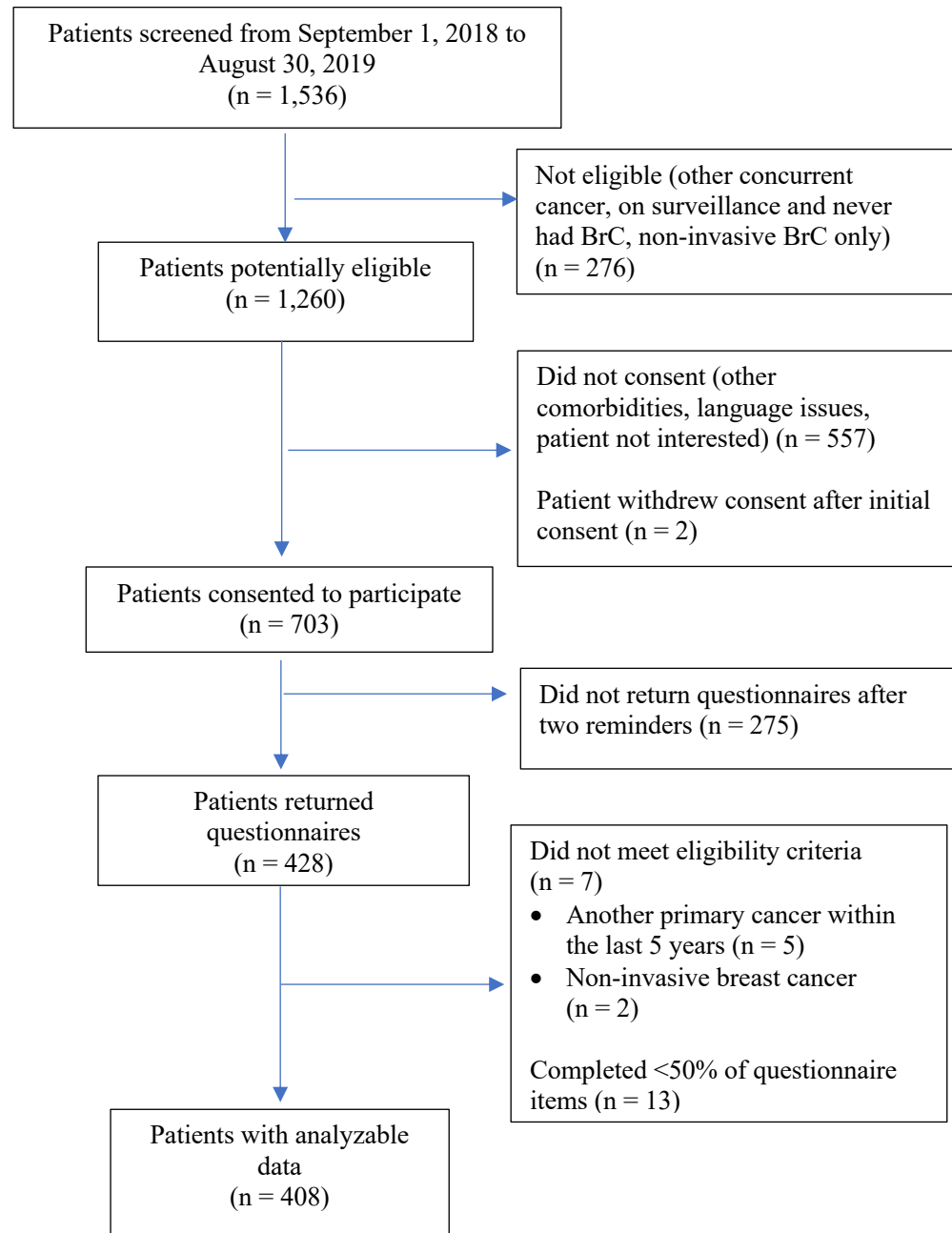

Supplement: S1 Fig — (PDF) [file pone.0262635.s001.pdf]
