# Supplementary material for: Developing the Breast Utility Instrument, a preference-based instrument to measure health-related quality of life in women with breast cancer: Confirmatory factor analysis of the EORTC QLQ-C30 and BR45 to establish dimensions
Source: PLoS One. 2022 Feb 4;17(2):e0262635. doi: 10.1371/journal.pone.0262635 (PMC8815914; doi:10.1371/journal.pone.0262635)
Supplement: S2 Fig — (PDF) [file pone.0262635.s002.pdf]

S2 Fig: Item response distributions of EORTC QLQ-C30 subscales by BrC health state

i) Physical functioning

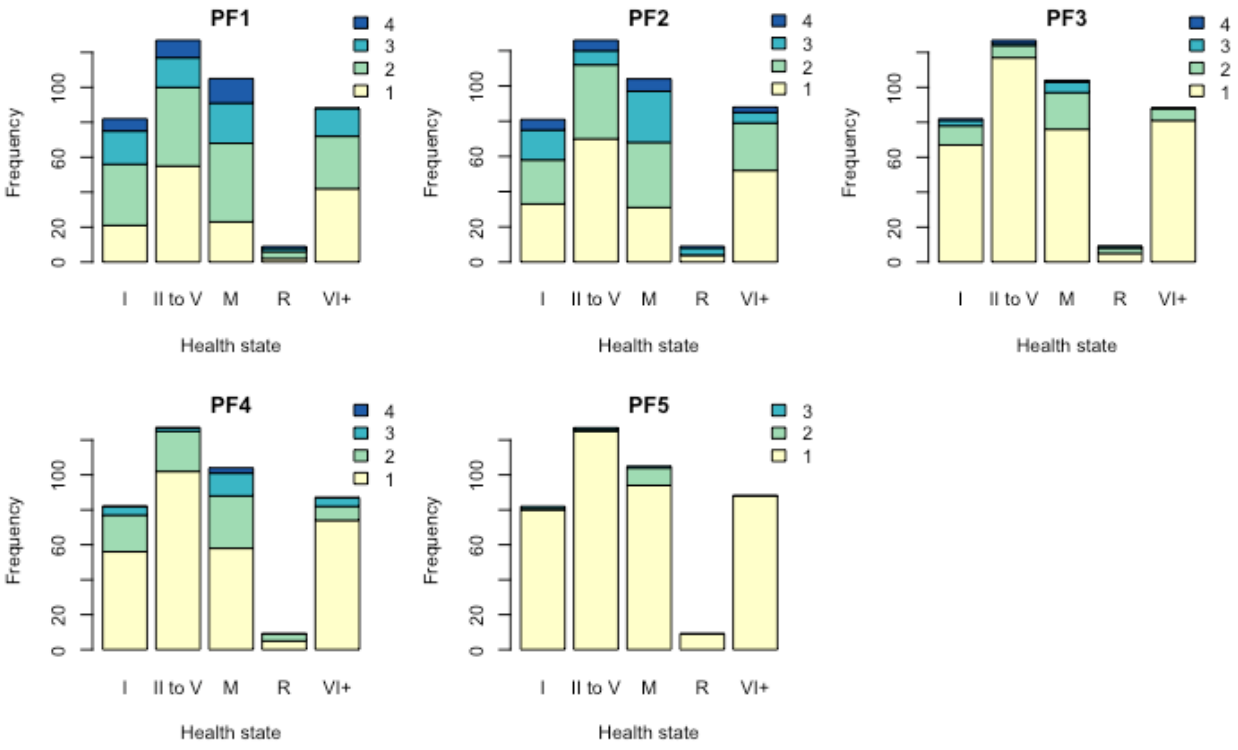

PF1. Do you have any trouble doing strenuous activities, like carrying a heavy shopping bag or a suitcase?
PF2. Do you have any trouble taking a long walk?
PF3. Do you have any trouble taking a short walk outside of the house?
PF4. Do you need to stay in bed or a chair during the day?
PF5. Do you need help with eating, dressing, washing yourself or using the toilet?

Health states:
I: first year after diagnosis of primary BrC;
R: first year after date of recurrence, or new primary BrC;
II-V: second to fifth year after primary BrC or recurrence treated with curative intent;
VI+: sixth and following years after a primary BrC or recurrence treated with curative intent;
M: metastatic BrC.

Response options range from 1 to 4:
1: Not at all
2: A little
3: Quite a bit
4. Very much

ii) Role functioning

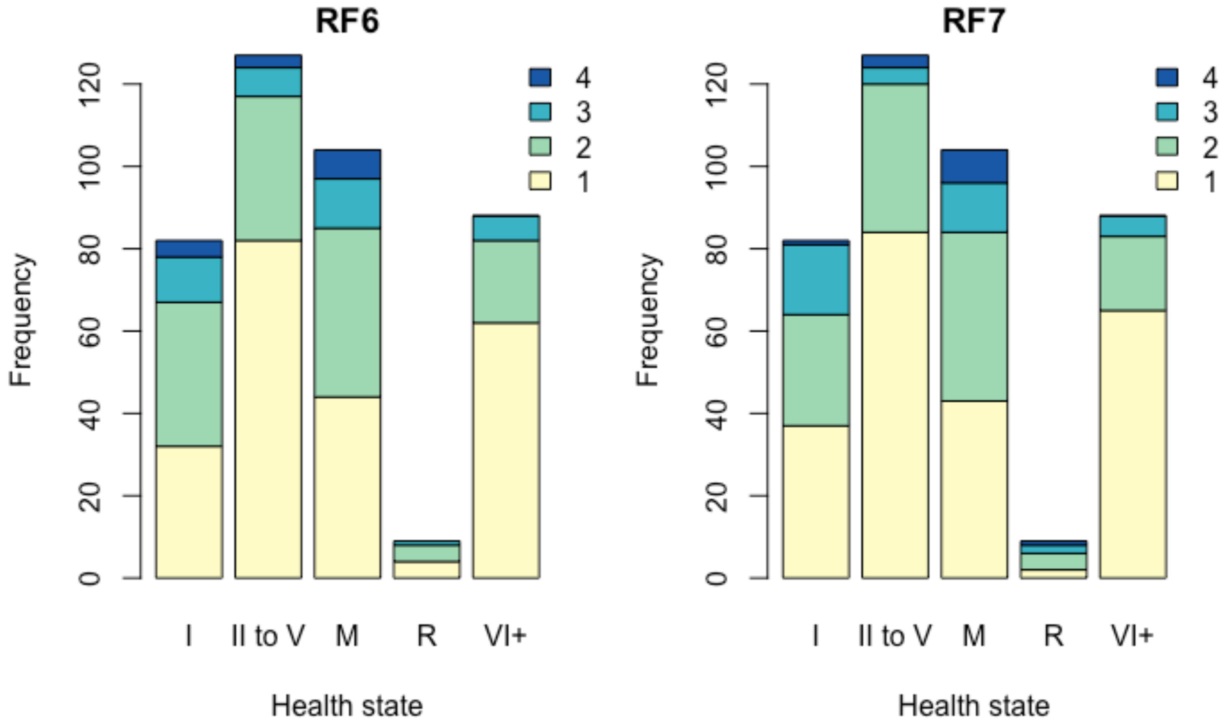

During the past week:

RF6. Were you limited in doing either your work or other daily activities?
RF7. Were you limited in pursuing your hobbies or other leisure time activities?

iii) Emotional functioning

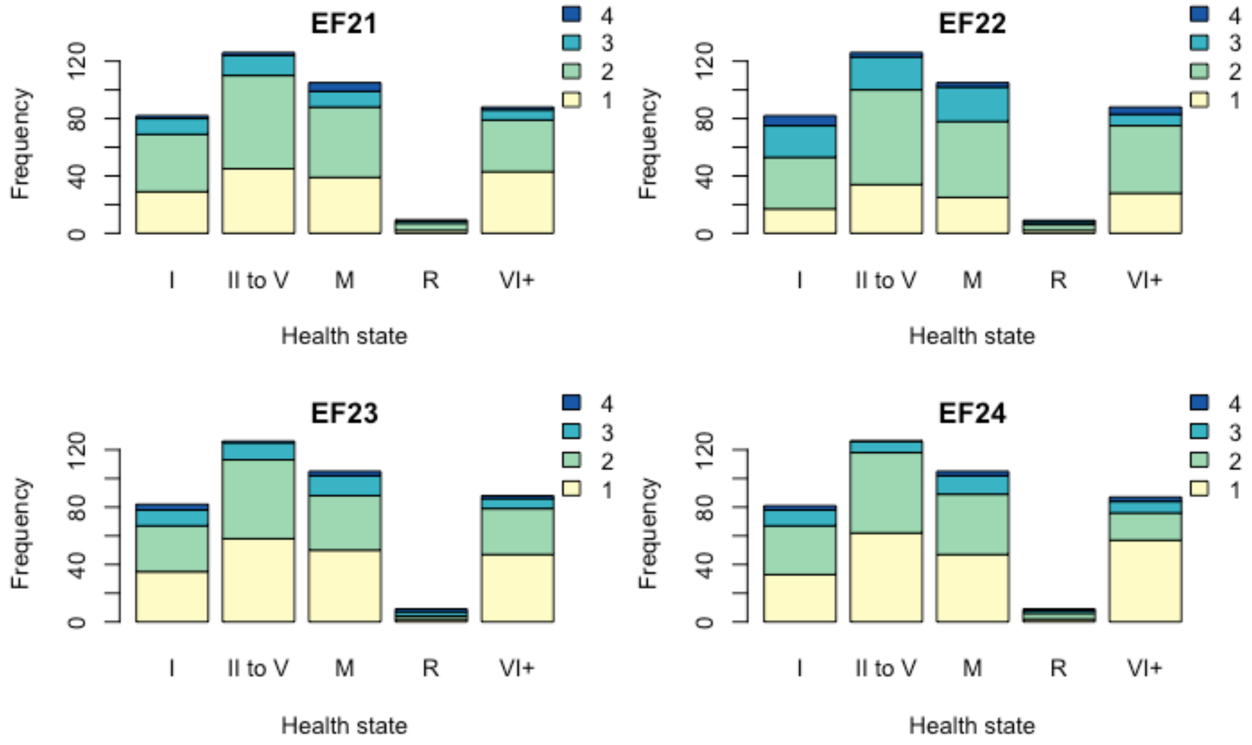

During the past week:

EF21. Did you feel tense?
EF22. Did you worry?
EF23. Did you feel irritable?
EF24. Did you feel depressed?

iv) **Cognitive functioning**

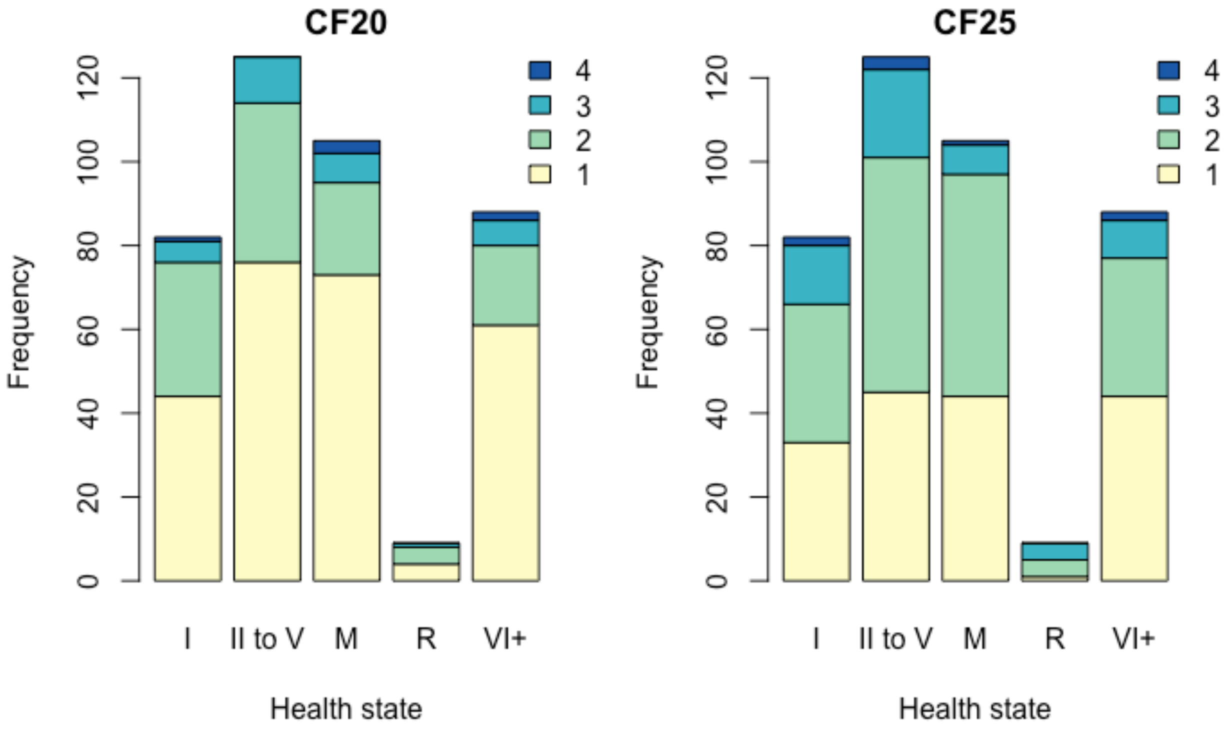

During the past week:

CF20. Have you had difficulty in concentrating on things, like reading a newspaper or watching television?
CF25. Have you had difficulty remembering things?

v) Social functioning

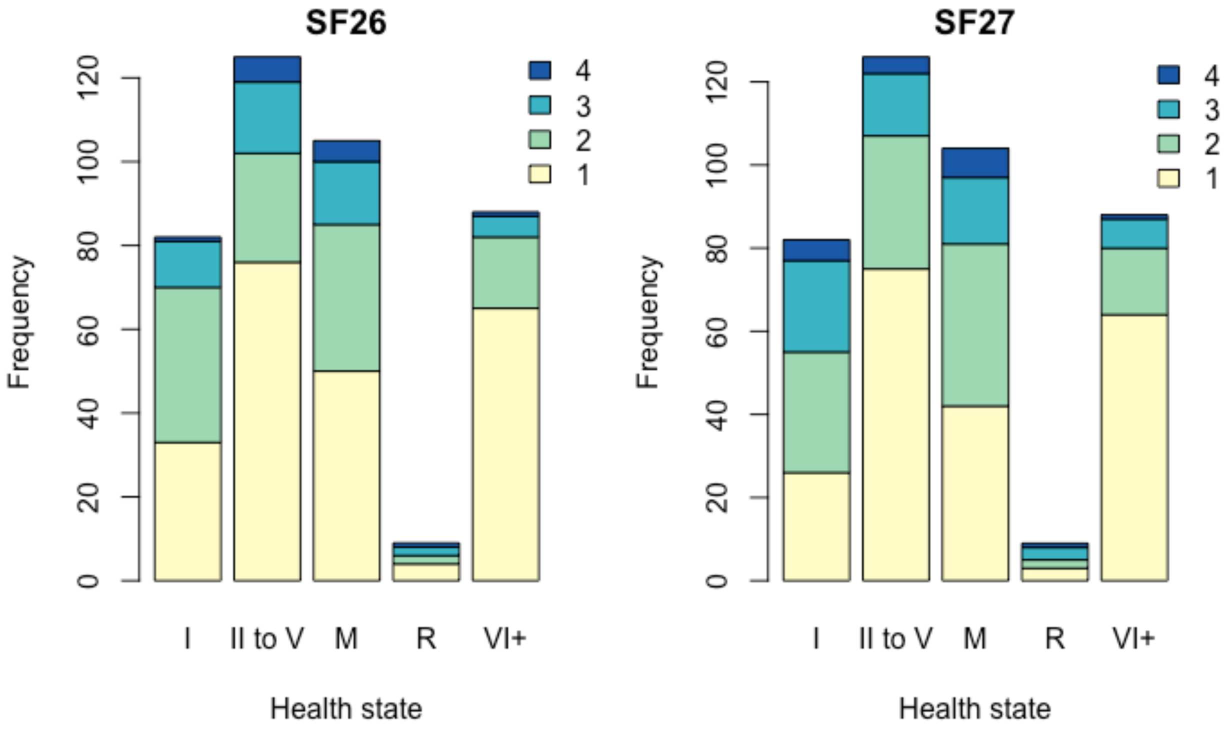

During the past week:

SF26. Has your physical condition or medical treatment interfered with your <u>family</u> life?
SF27. Has your physical condition or medical treatment interfered with your <u>social</u> activities?

vi) Fatigue symptom

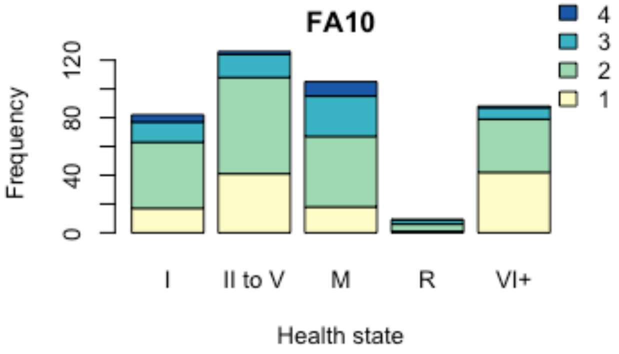

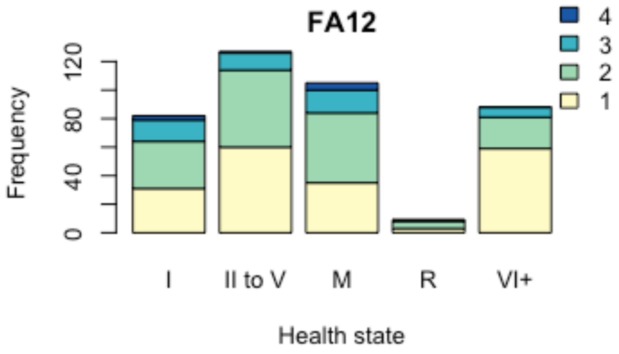

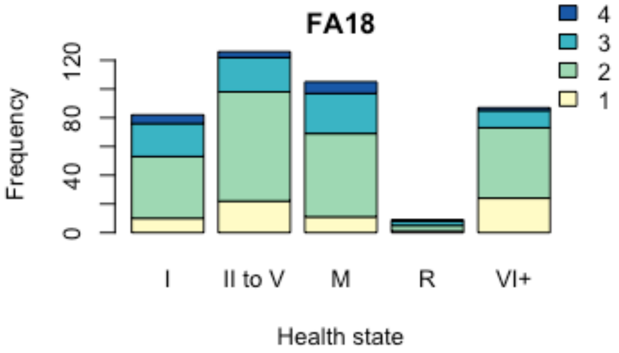

During the past week:

FA10. Did you need to rest?
FA12. Have you felt weak?
FA18. Were you tired?

vii) Pain symptom domain

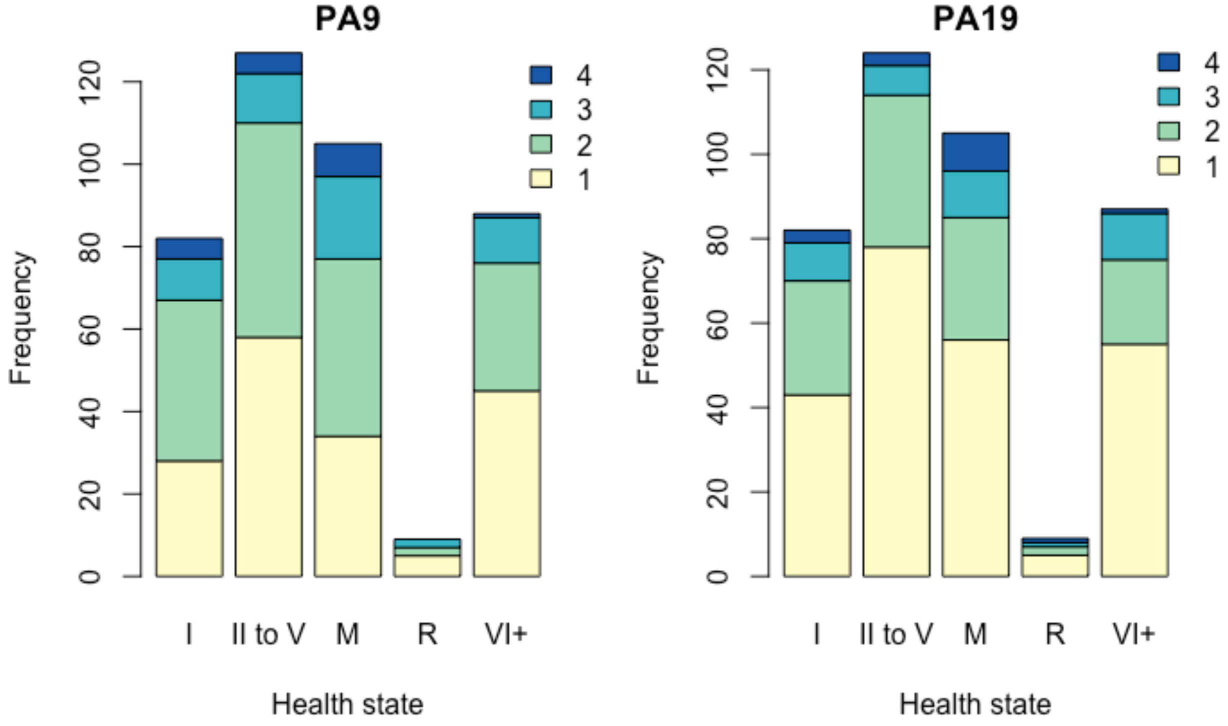

During the past week:

PA9. Have you had pain?
PA19. Did pain interfere with your daily activities?

viii) Nausea, vomiting, constipation, and diarrhea

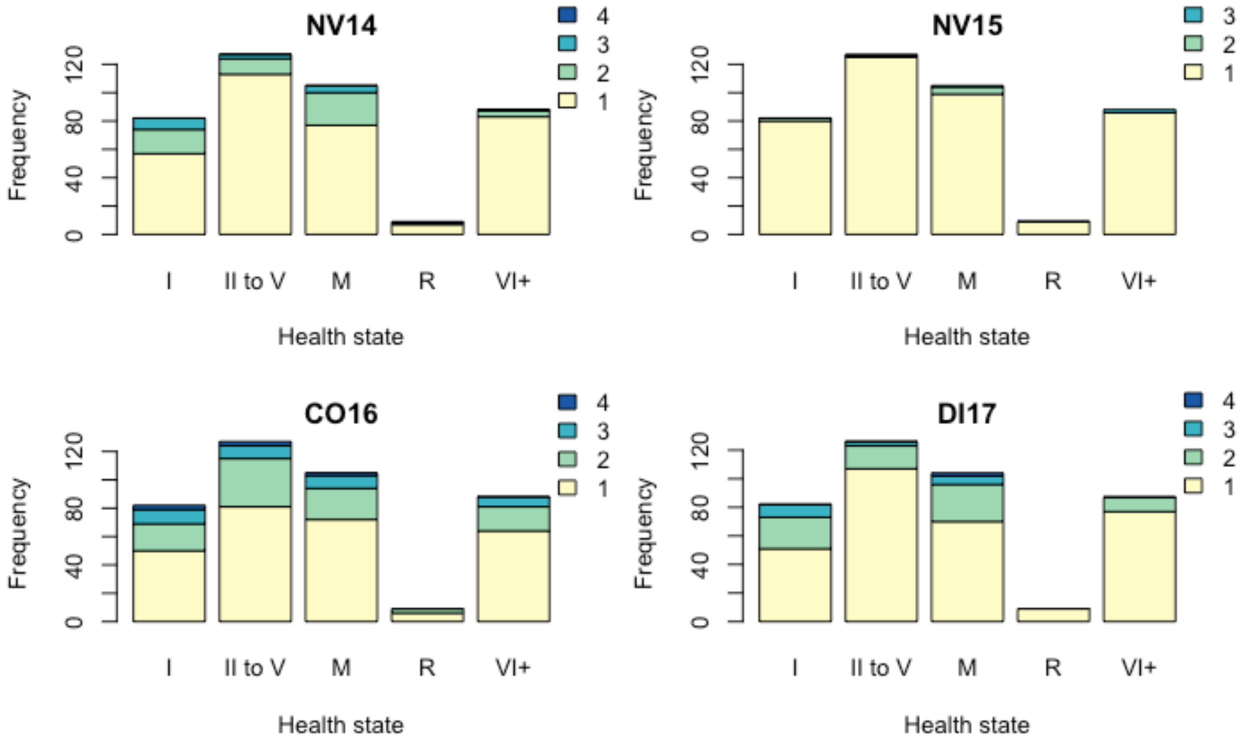

During the past week:

NV14: Have you felt nauseated?
NV15: Have you vomited?
CO16: Have you been constipated?
DI17: Have you had diarrhea?
