# Supplementary material for: Developing the Breast Utility Instrument, a preference-based instrument to measure health-related quality of life in women with breast cancer: Confirmatory factor analysis of the EORTC QLQ-C30 and BR45 to establish dimensions
Source: PLoS One. 2022 Feb 4;17(2):e0262635. doi: 10.1371/journal.pone.0262635 (PMC8815914; doi:10.1371/journal.pone.0262635)
Supplement: S3 Fig — (PDF) [file pone.0262635.s003.pdf]

S3 Fig: Item response distributions on EORTC QLQ BR45 subscales by BrC health state

ix) Body image

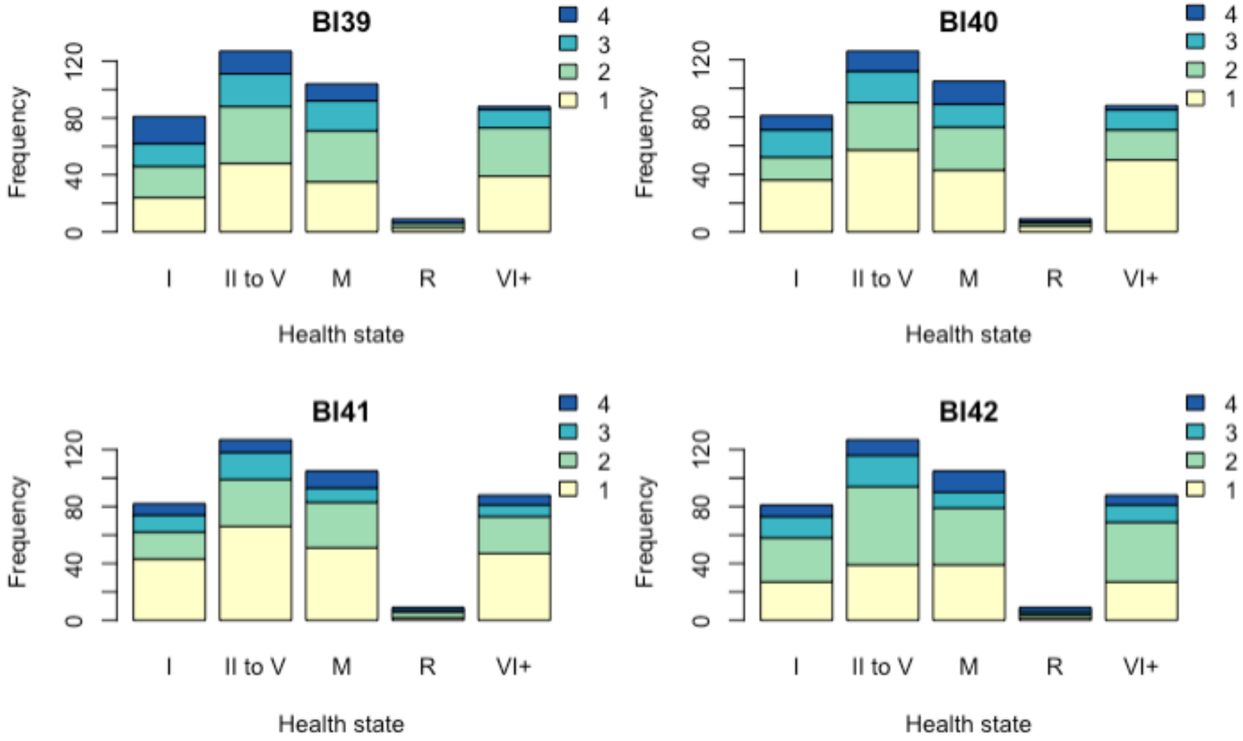

During the past week:

BI39: Have you felt physically less attractive as a result of your disease or treatment?
BI40: Have you felt less feminine as a result of your disease or treatment?
BI41: Have you had problems looking at yourself naked?
BI42: Have you been dissatisfied with your body?

x) **Sexual functioning and enjoyment**

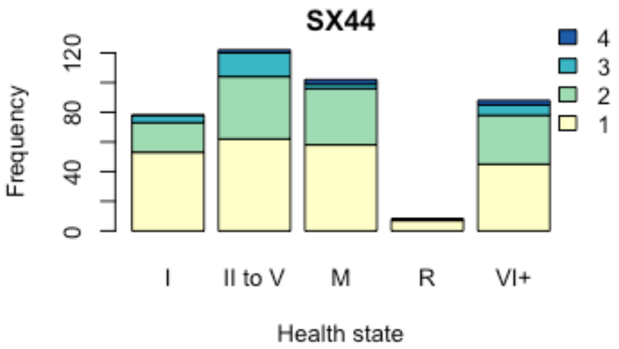

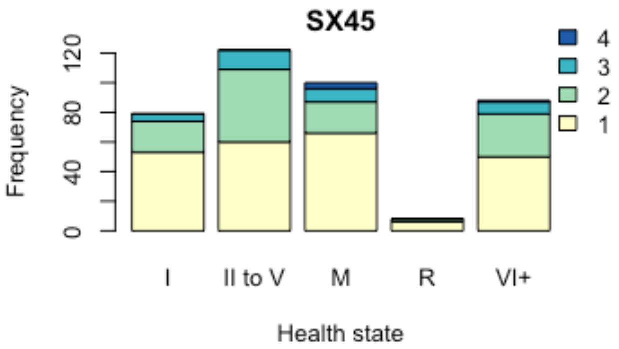

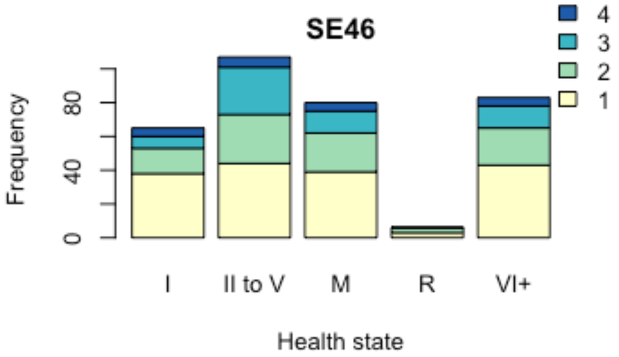

During the past four weeks:

SX44: Have you been interested in sex?
SX45: Have you been sexually active (with or without intercourse)?
SE46: Has sex been enjoyable for you?

xi) **Breast satisfaction**

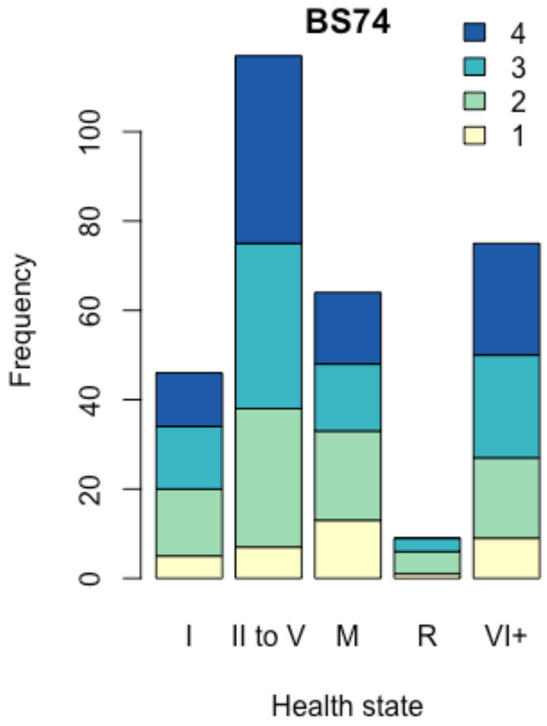

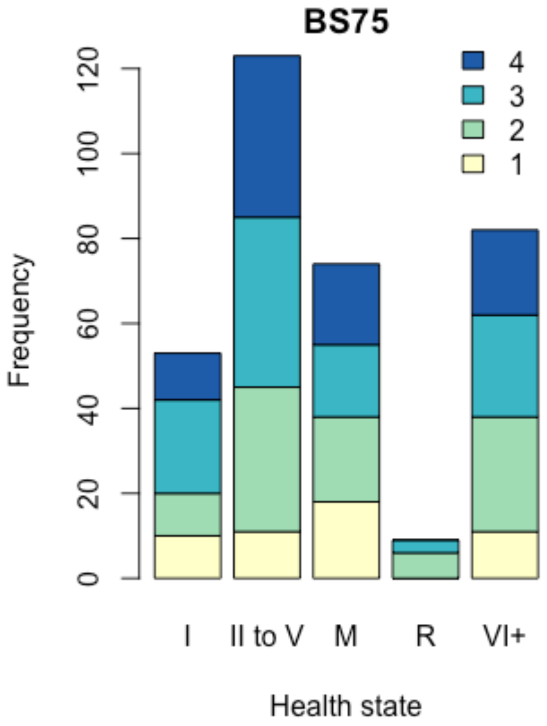

During the past week:

BS74: Have you been satisfied with the cosmetic result of surgery?
BS75: Have you been satisfied with the appearance of the skin of your affected breast (thoracic area)?

xii) Systemic therapy side effects

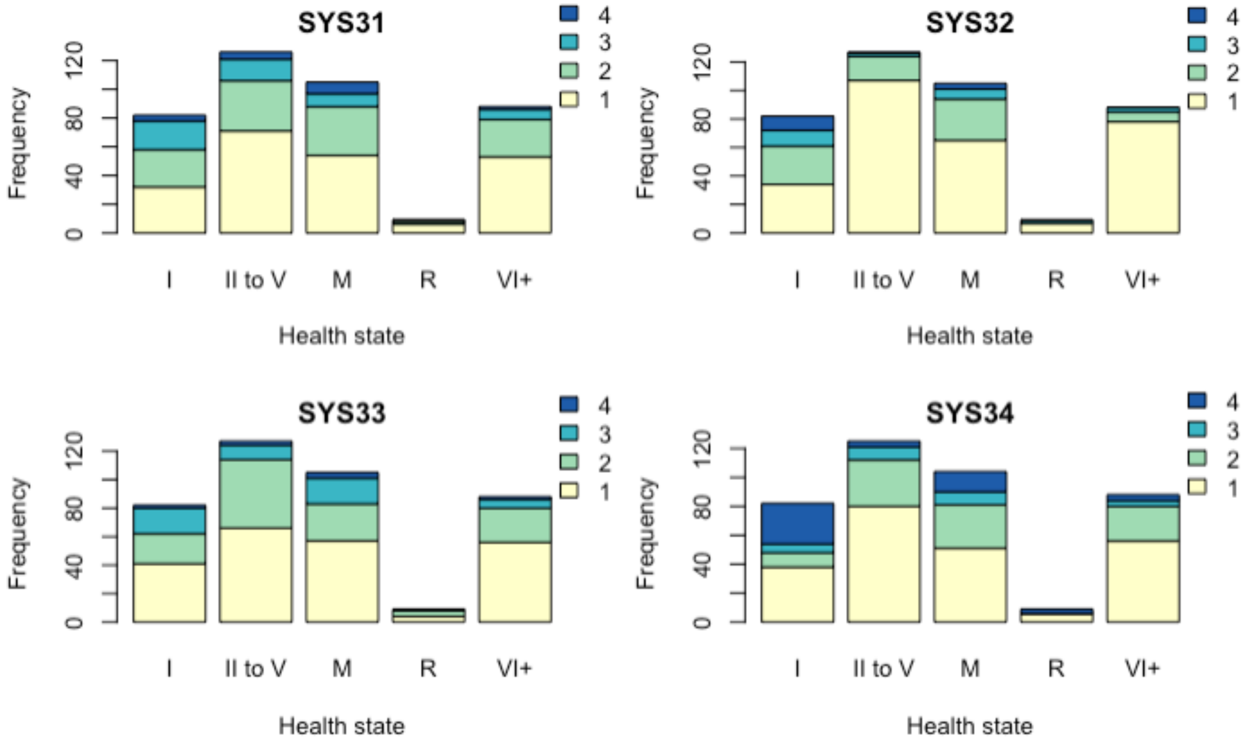

During the past week:

SYS31: Have you had dry mouth?
SYS32: Have food and drink tasted differed than usual?
SYS33: Have your eyes been painful, irritated or watery?
SYS34: Have you lost any hair?

xii) Systemic therapy side effects /cont…

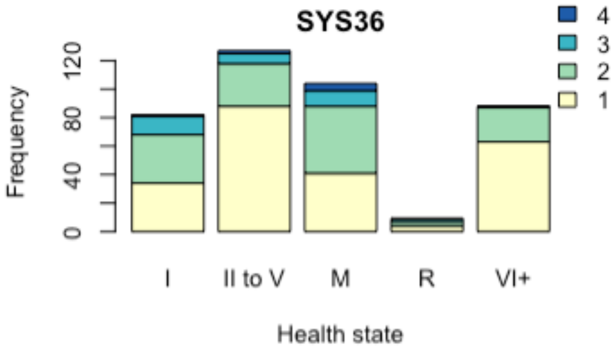

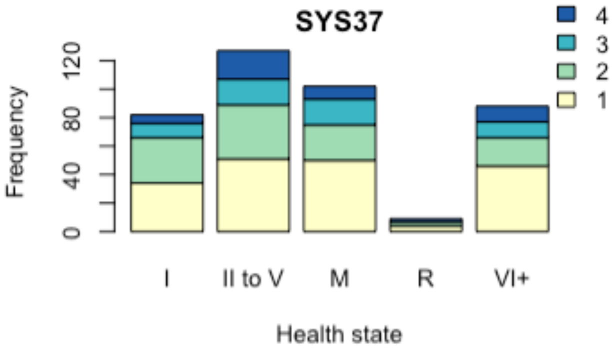

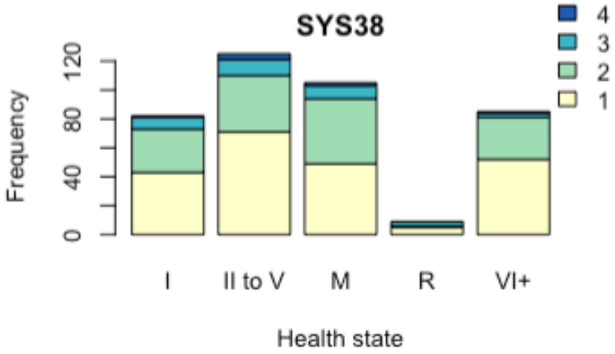

During the past week:

SYS36: Have you felt ill or unwell?
SYS37: Have you had hot flushes?
SYS38: Have you had headaches?

xiii) Arm symptoms

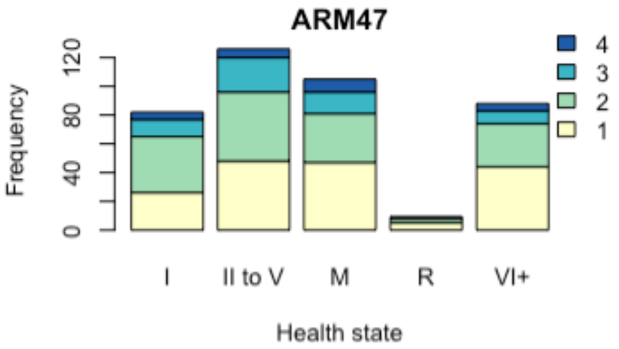

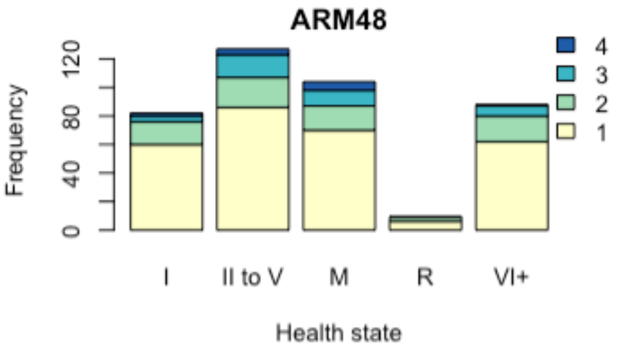

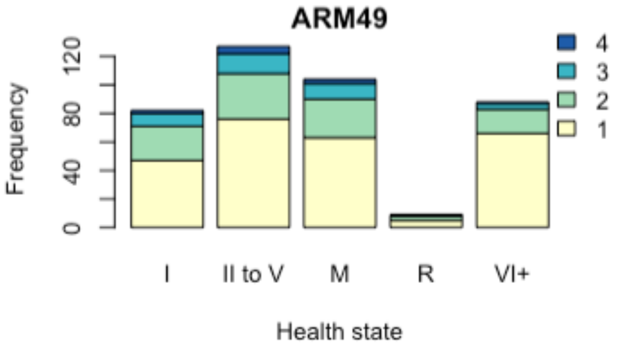

During the past week:

ARM47: Have you had any pain in your arm or shoulder?
ARM48: Have you had a swollen arm or hand?
ARM49: Have you had any problems raising your arm or moving it sideways?

xiv) Breast symptoms

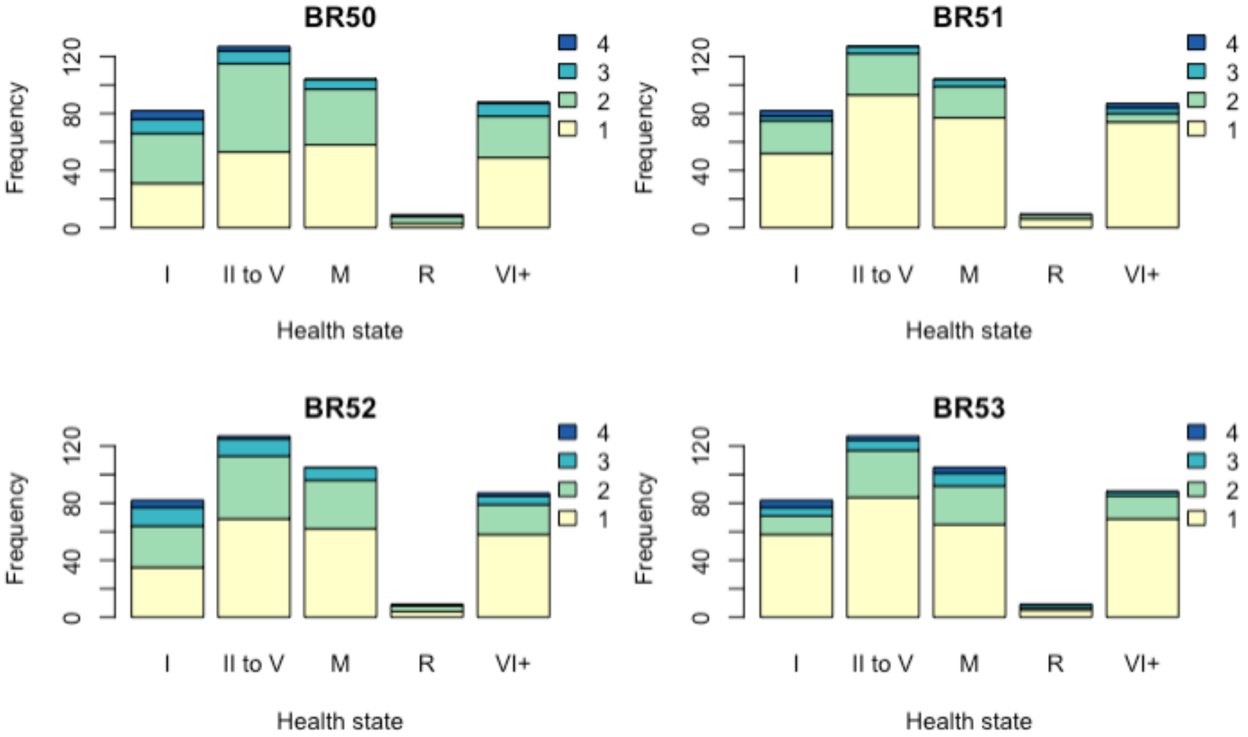

During the past week:

BR50: Have you had any pain in the area of your affected breast?
BR51: Has the area of your affected breast been swollen?
BR52: Has the area of your affected breast been oversensitive?
BR53: Have you had skin problems on or in the area of your affected breast (e.g., itchy, dry, flaky)?

xv) Skin mucositis

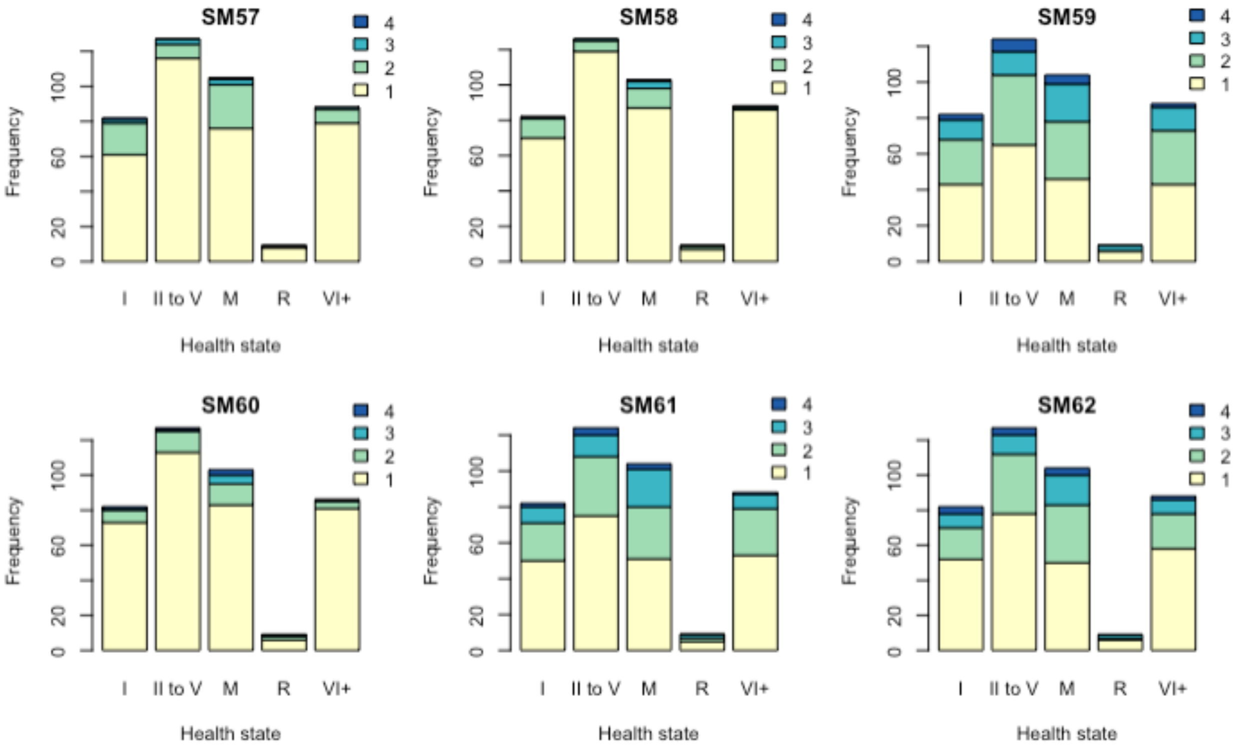

During the past week:

SM57: Have you had soreness in your mouth?
SM58: Have you had any redness in your mouth?
SM59: Have you had any pain in your hands or feet?
SM60: Have you had any redness on your hands or feet?
SM61: Have you had tingling in your fingers or toes?
SM62: Have you had numbness in your fingers or toes?

xvi) Endocrine therapy symptoms

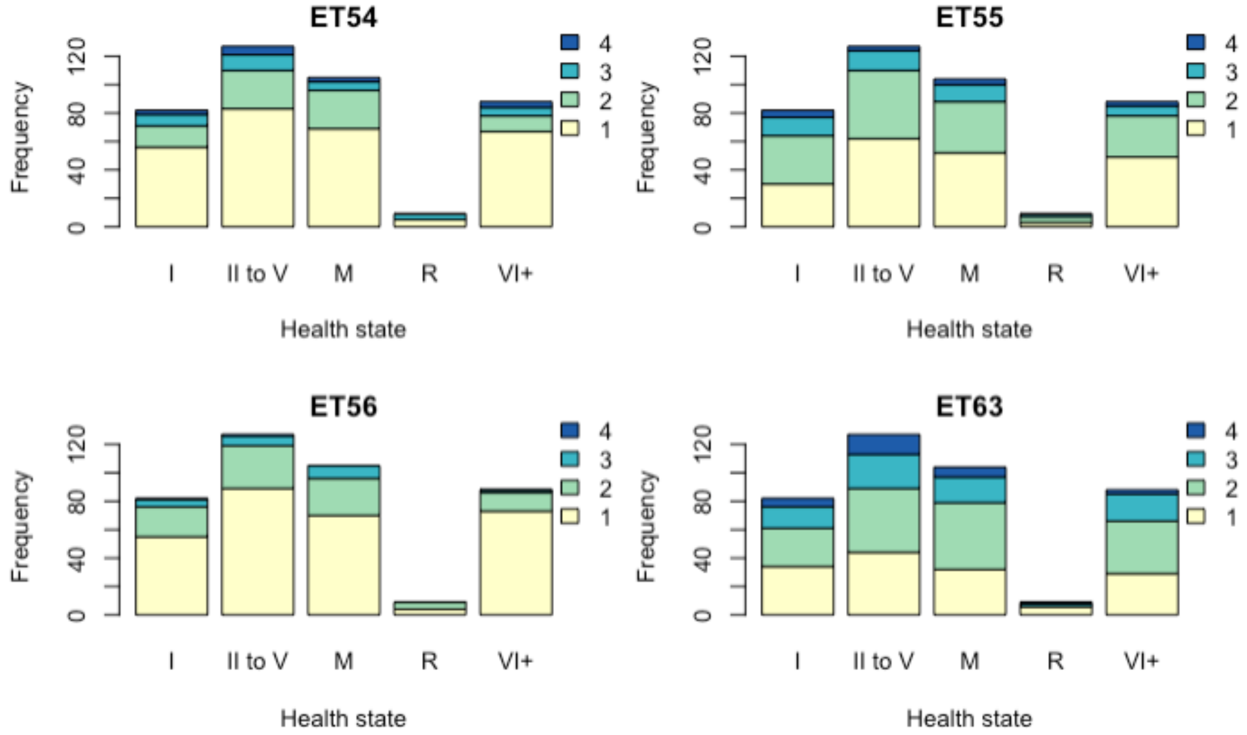

During the past week:

ET54: Have you sweated excessively?
ET55: Have you had mood swings?
ET56: Have you been dizzy?
ET63: Have you had problems with your joints?

xvi) Endocrine therapy symptoms /cont…

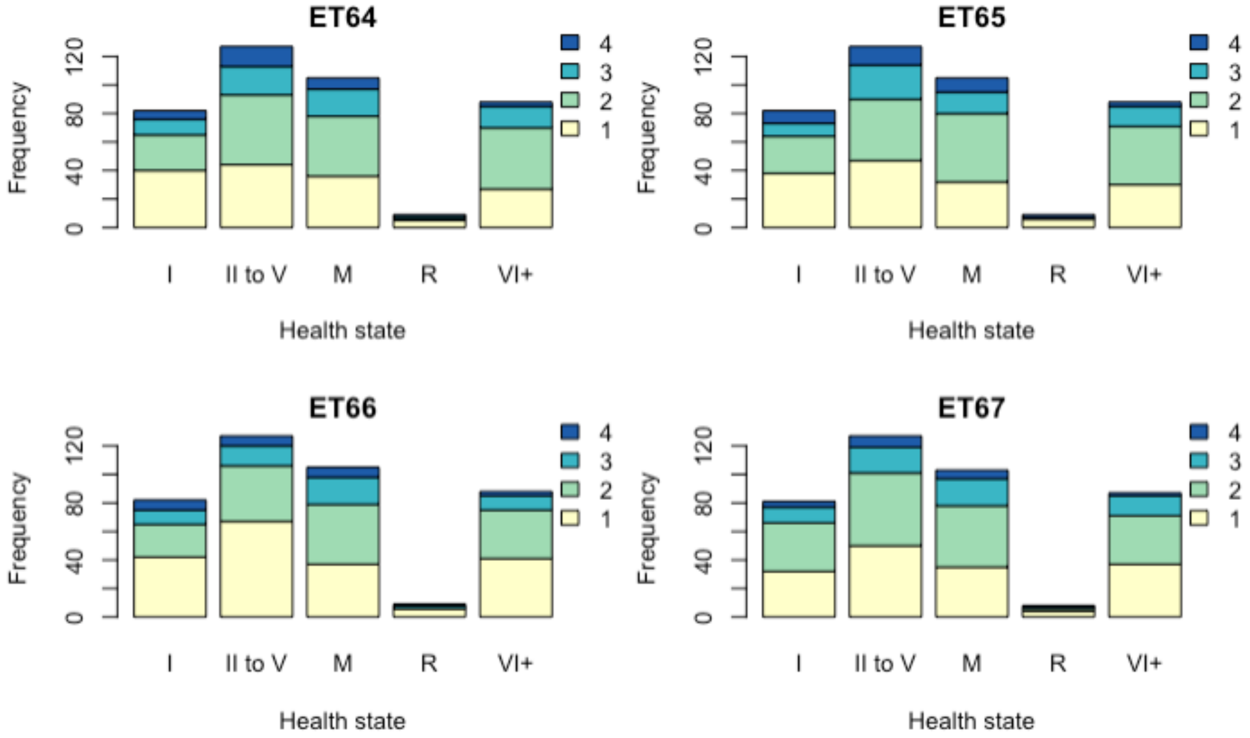

During the past week:

ET64: Have you had stiffness in your joints?
ET65: Have you had pain in your joints?
ET66: Have you had aches or pains in your bones?
ET67: Have you had aches or pains in your muscles?

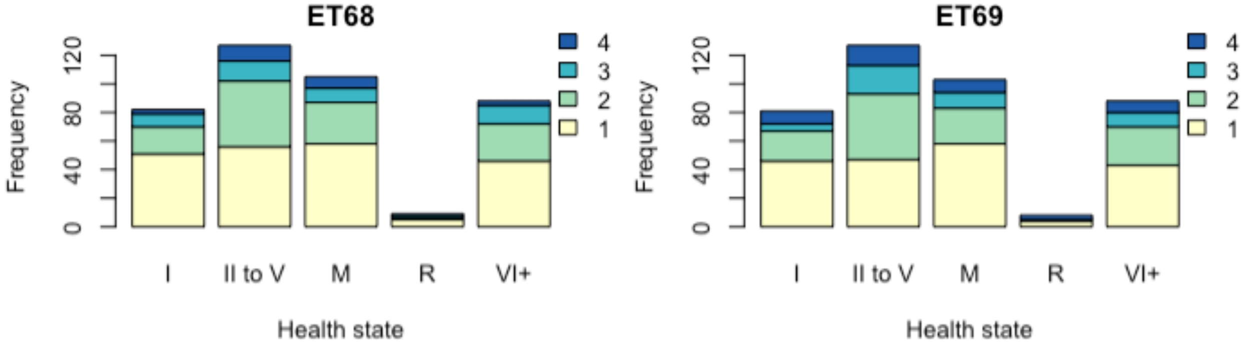

During the past week:

ET68: Have you gained weight?
ET69: Has weight gain been a problem for you?
