# Supplementary material for: Developing the Breast Utility Instrument, a preference-based instrument to measure health-related quality of life in women with breast cancer: Confirmatory factor analysis of the EORTC QLQ-C30 and BR45 to establish dimensions
Source: PLoS One. 2022 Feb 4;17(2):e0262635. doi: 10.1371/journal.pone.0262635 (PMC8815914; doi:10.1371/journal.pone.0262635)
Supplement: S4 Fig — (PDF) [file pone.0262635.s004.pdf]

S4 Fig: Inter-subscale correlations proportional to colour intensity and dot size.

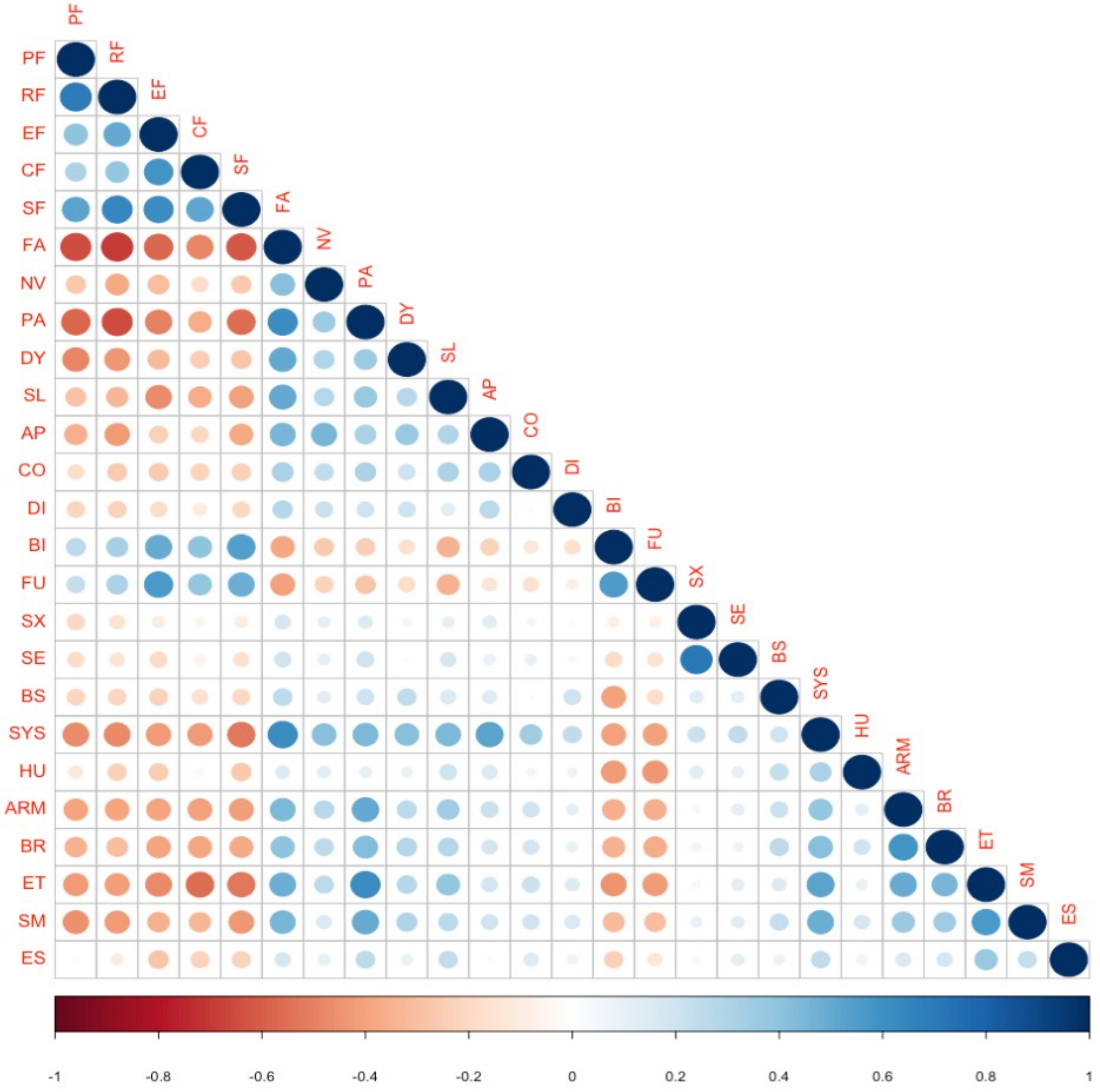

All EORTC QLQ-C30 and BR45 subscales are listed on the x and y axes. The inter subscale-scale correlations are represented by a continuum from dark blue (+1) to dark red (-1). Higher scores for symptoms imply more severe symptoms, while higher scores for functioning imply greater ability; therefore darker coloured correlations represent stronger correlations.

PF: physical function, RF: role function, EF: emotional function, CF: cognitive function, SF: social function, FA: fatigue, NV: nausea and vomiting, PA: pain, DY: dyspnea, SL: insomnia, AP: appetite loss, CO: constipation, DI: diarrhea, BI: body image, SX: sexual functioning, SE: sexual enjoyment, BS: breast satisfaction, SYS: systemic therapy side effects, HU: upset by hair loss, ARM: arm symptoms, BR: breast symptoms, ET: endocrine therapy symptoms, SM: skin mucositis symptoms, ES: endocrine sexual symptoms
