# Supplementary material for: Developing the Breast Utility Instrument, a preference-based instrument to measure health-related quality of life in women with breast cancer: Confirmatory factor analysis of the EORTC QLQ-C30 and BR45 to establish dimensions
Source: PLoS One. 2022 Feb 4;17(2):e0262635. doi: 10.1371/journal.pone.0262635 (PMC8815914; doi:10.1371/journal.pone.0262635)
Supplement: S1 Table — (PDF) [file pone.0262635.s005.pdf]

S1 Table: QLQ-C30 and BR45 items, associated scale, percentage missing

Item	Item stem wording	Scale	Percentage missing (%)
**EORTC QLQC30**			
1	Trouble doing strenuous activities.	PF	0.0
2	Trouble taking a long walk.	PF	0.7
3	Trouble taking a short walk.	PF	0.2
4	Need to stay in bed or a chair during the day.	PF	0.5
5	Need help with eating, dressing, washing yourself or using the toilet.	PF	0.0
During the past week:			
6	Limited in doing either your work or other daily activities.	RF	0.2
7	Limited in pursuing your hobbies or other leisure time activities.	RF	0.2
8	Short of breath.	DY	0.0
9	Had pain.	PA	0.0
10	Need to rest.	FA	0.2
11	Trouble sleeping.	SL	0.0
12	Felt weak.	FA	0.0
13	Lacked appetite.	AP	0.0
14	Felt nauseated.	NV	0.0
15	Vomited.	NV	0.0
16	Constipated.	CO	0.0
17	Had diarrhea.	DI	0.7
18	Tired.	FA	0.5
19	Pain interfered with your daily activities.	PA	1.0
20	Difficulty in concentrating on things, like reading a newspaper or watching television.	CF	0.5
21	Felt tense.	EF	0.2
22	Worry.	EF	0.2
23	Felt irritable.	EF	0.2
24	Felt depressed.	EF	0.7
25	Difficulty remembering things.	CF	0.5
26	Physical condition or medical treatment interfered with your family life.	SF	0.5
27	Physical condition or medical treatment interfered with your social activities.	SF	0.5
28	Physical condition or medical treatment caused you financial difficulties.	FI	0.7
29	Rate your overall health during the past week.	QL	0.5
30	Rate your overall quality of life during the past week.	QL	0.5

**EORTC QLQ BR45**			
During the past week:			
31	Had a dry mouth.	SYS	0.2
32	Food and drink tasted different than usual.	SYS	0.0
33	Eyes been painful, irritated or watery.	SYS	0.0
34	Lost any hair.	SYS	0.7
35	Answer this question only if you have lost any hair:<br>Upset by the loss of your hair.	HU	49.5
36	Felt ill or unwell.	SYS	0.2
37	Hot flushes.	SYS	0.7
38	Had headaches.	SYS	1.2
39	Felt physically less attractive as a result of your disease or treatment.	BI	0.5
40	Felt less feminine as a result of your disease or treatment.	BI	0.5
41	Problems looking at yourself naked.	BI	0.0
42	Dissatisfied with your body.	BI	0.2
43	Worried about your health in the future.	FU	0.0
During the past four weeks:			
44	Interested in sex.	SX	3.2
45	Sexually active (with or without intercourse).	SX	3.4
46	Sex been enjoyable.	SE	17
During the past week:			
47	Pain in your arm or shoulder.	ARM	0.2
48	Swollen arm or hand.	ARM	0.2
49	Problems raising your arm or moving it sideways.	ARM	0.2
50	Have you had any pain in the area of your<br>affected breast?	BR	0.2
51	Area of your affected breast been swollen.	BR	0.5
52	Area of your affected breast been oversensitive.	BR	0.2
53	Skin problems on or in the area of your affected breast (e.g., itchy, dry,<br>flaky).	BR	0.0
54	Sweated excessively.	ET	0.0
55	Had mood swings.	ET	0.2
56	Dizzy.	ET	0.0
57	Soreness in your mouth.	SM	0.0
58	Redness in your mouth.	SM	0.7
59	Pain in your hands or feet.	SM	1.0
60	Redness on your hands or feet.	SM	1.0
61	Tingling in your fingers or toes.	SM	1.0
62	Numbness in your fingers or toes.	SM	0.2
63	Problems with your joints.	ET	0.2
64	Stiffness in your joints.	ET	0.0
65	Pain in your joints.	ET	0.0
66	Aches or pains in your bones.	ET	0.0
67	Aches or pains in your muscles.	ET	1.2

68	Gained weight.	ET	0.0
69	Weight gain been a problem for you.	ET	1.0
During the past four weeks:			
70	Had dry vagina.	ES	1.9
71	Discomfort in your vagina.	ES	1.0
Answer the following two questions only if you have been sexually active:			
72	Pain in your vagina during sexual activity.	ES	50.7
73	Experienced a dry vagina during sexual activity.	ES	51.5
During the past week:			
74	Satisfied with the cosmetic result of the surgery.	BS	24.3
75	Satisfied with the appearance of the skin of your affected breast (thoracic area).	BS	17.0
